# Phylogeographic Analysis of *Mycobacterium kansasii* Isolates from Patients with *M. kansasii* Lung Disease in Industrialized City, Taiwan

**DOI:** 10.3201/eid3008.240021

**Published:** 2024-08

**Authors:** Patrick George Tobias Cudahy, Po-Chen Liu, Joshua L. Warren, Benjamin Sobkowiak, Chongguang Yang, Thomas R. Ioerger, Chieh-Yin Wu, Po-Liang Lu, Jann-Yuan Wang, Hsiao-Han Chang, Hung-Ling Huang, Ted Cohen, Hsien-Ho Lin

**Affiliations:** Yale School of Medicine, New Haven, Connecticut, USA (P.G.T. Cudahy);; National Taiwan University, Taipei, Taiwan (P.-C. Liu, C.-Y. Wu, J.-Y. Wang, H.-H. Lin);; Yale School of Public Health, New Haven (J.L. Warren, B. Sobkowiak, T. Cohen);; Sun Yat-Sen University School of Public Health, Guangzhou, China (C. Yang);; Texas A&M University, College Station, Texas, USA (T.R. Ioerger);; Kaohsiung Medical University, Kaohsiung, Taiwan (P.-L. Lu, H.-L. Huang);; Kaohsiung Medical University Hospital, Kaohsiung (P.-L. Lu, H.-L. Huang);; National Tsing Hua University, Hsinchu, Taiwan (H.-H. Chang);; Kaohsiung Municipal Ta-Tung Hospital, Kaohsiung (H.-L. Huang)

**Keywords:** tuberculosis and other mycobacteria, bacteria, environmental exposures, genetic relatedness, *Mycobacterium kansasii*, transmission, whole-genome sequencing, Taiwan

## Abstract

Little is known about environmental transmission of *Mycobacterium kansasii*. We retrospectively investigated potential environmental acquisition, primarily water sources, of *M. kansasii* among 216 patients with pulmonary disease from an industrial city in Taiwan during 2015–2017. We analyzed sputum mycobacterial cultures using whole-genome sequencing and used hierarchical Bayesian spatial network methods to evaluate risk factors for genetic relatedness of *M. kansasii* strains. The mean age of participants was 67 years; 24.1% had previously had tuberculosis. We found that persons from districts served by 2 water purification plants were at higher risk of being infected with genetically related *M. kansasii* isolates. The adjusted odds ratios were 1.81 (1.25–2.60) for the Weng Park plant and 1.39 (1.12–1.71) for the Fongshan plant. Those findings unveiled the association between water purification plants and *M. kansasii* pulmonary disease, highlighting the need for further environmental investigations to evaluate the risk for *M. kansasii* transmission.

*Mycobacterium kansasii*, a slow-growing nontuberculous mycobacteria (NTM), can cause destructive pulmonary diseases in humans that cause similar clinical manifestations to those of *M. tuberculosis* pulmonary disease (*1*). In recent years, *M. kansasii* has become one of the most frequently reported NTMs in the world (*2*), but whether increasing notifications in some countries (*3*) reflects true increases in the incidence of *M. kansasii* disease or improvements in laboratory identification is not well understood.

Infection with *M. kansasii* has been predominantly reported in urban settings, in high-density and low-income communities, and among gold miners (*4*). Despite recent reports of potential human-to-human transmission for other NTMs (*5*), *M. kansasii* lung disease has been generally assumed to be acquired from environmental sources (*6*). Nonetheless, the precise route of transmission is yet to be characterized. *M. kansasii* is ubiquitous in the environment; tap water is reported as a major reservoir (*6*). The waterborne acquisition of *M. kansasii* is enabled by its intrinsic resistance to disinfectants, acid, and heat (*7*); its ability to survive in oligotrophic water; and its ability to form pipe surface biofilms (*8*). In previous studies, *M. kansasii* has been isolated from the water distribution system in the same communities in which cases with *M. kansasii* disease arise (*9*). However, evidence on environmental acquisition on the basis of genotyping of clinical or environmental isolates has been limited.

We investigated the environmental acquisition of *M. kansasii* in the industrial city of Kaohsiung, located in southern Taiwan. Our earlier multicenter study revealed that the number of patients with *M. kansasii* pulmonary infection was nearly 5-fold higher in Kaohsiung than in Taipei in northern Taiwan (*10*). Higher humidity levels, warmer temperatures, and industrial areas with greater air pollution in Kaohsiung might explain this difference. We previously identified 2 spatial hotspots of high risk for *M. kansasii* infection in Kaohsiung by using clinical data collected from patients in all tertiary medical centers of the city (*11*). We hypothesized that specific water supplies or heavy industrial areas might be associated with the risk for *M. kansasii* infection. In this study, we extended the previous spatial analysis to include whole-genome sequencing (WGS) data of clinical *M. kansasii* isolates along with geographic information from patients, area-level industrialization, and water supply systems to comprehensively assess the risk and drivers of *M. kansasii* in Kaohsiung.

## Methods

### Study Participants

We included 302 patients >20 years of age who had newly diagnosed *M. kansasii* lung disease, consistent with American Thoracic Society/Infectious Diseases Society of America guidelines (*12*) from 1 tertiary medical center and its affiliated regional hospitals in Kaohsiung during 2015–2017. We conducted chart reviews to collect clinical and demographic data from eligible persons.

### Mycobacterial WGS

Of the 302 patients, we performed WGS on mycobacterial isolates from 243 patients with high-quality culture samples from pretreatment sputum or bronchoalveolar lavage fluid. We performed sequencing in the Laboratory of Genomics and Bioinformatics Service at Texas A&M AgriLife (College Station, TX, USA) and constructed libraries by using the NEXTFLEX Rapid XP DNA-Seq Kit (Revvity, https://www.revvity.com). We performed paired-end sequencing using the NovaSeq 6000 Sequencing System (Illumina, https://www.illumina.com) with a read length of 150 bp and anticipated minimum mean depth of coverage of 100×. We removed 22 samples with most reads from a species other than *M. kansasii* from analysis. One additional sample had a large degree of nonmycobacterial reads, and 4 samples were from participants missing clinical or residential data. We excluded those from analysis, leaving 216 samples for our primary analysis.

### Environmental Exposure Data

**Figure 1:**
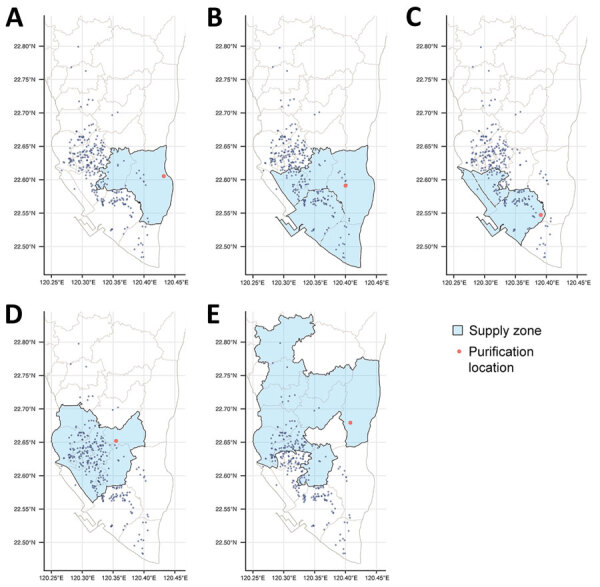
Spatial distribution of residential addresses of *Mycobacterium kansasii* isolates from patients, water purification plants, and corresponding supply zones in phylogeographic analysis of *M. kansasii* isolates from patients with *M. kansasii* lung disease in industrialized city, Taiwan. Spatial distribution is shown for purification plants in Weng Park (A), Kaotan (B), Fongshan (C), Chengcing (D), and Pingding (E). Cases are indicated by blue dots, plants by red dots, and supply zones by light blue regions. Gray line represent the district boundaries.

Our primary environmental exposure of interest was the water sources. We collected information on the service areas of water purification plants in Kaohsiung through the official website of Taiwan Water Corporation (*13*). In total, 5 water purification plants provide service to most households in Kaohsiung. Among those, Pingding is the largest by volume (44% of the total water supply from the 5 plants), followed by Chengcing (29%), Kaotan (12%), Fongshan (10%), and Weng Park (4%) (*13*). The water supply networks of those purification plants overlap; a single district often receives service from >1 water plant (Figure 1). We identified areas of heavy industrial zoning in Kaohsiung and used a probabilistic approach to identify participants who had a high probability of working in 1 of those zones (Appendix).

### Data Analysis

We classified and filtered raw fastq reads using kraken2 (*14*) with a custom database, bracken (*15*), and ntm-profiler (*16*) (Appendix). We used Shovill (*17*) for de novo assembly using SPAdes (*18*). We aligned assemblies using ska (S.R. Harris, unpub. data, https://www.biorxiv.org/content/10.1101/453142v1) to the *M. kansasii* strain ATCC 12478, and all had >90% genomic coverage of the reference. We identified and masked regions of horizontal sequence transfer using Gubbins (*19*) and constructed a recombination-masked pairwise single-nucleotide polymorphism (SNP) distance matrix with snp-dists (*20*). We used this structure to create a phylogenetic tree with RAxML-NG (*21*) using the *M. kansasii* strain FDA-ARGOS 1615 as an outgroup, a generalized time reversible with gamma model of rate heterogeneity, and 50 starting trees (25 random and 25 parsimony) and calculated the transfer bootstrap expectation metric. To assess the association between genetic clade and water purification plant, we used Pearson χ^2^ tests.

To assess the sensitivity of our phylogenetic analysis, we repeated the analysis with the addition of genomic sequences obtained from public sources and identified as obtained from outside of Taiwan. We used a list of available isolates (*9*) to download short-read sequences of 22 specimens from the National Center for Biotechnology Information Sequence Read Archive database. We speciated and filtered the sequences with the same analysis pipeline as our samples from Taiwan and repeated phylogenetic analyses using a tree constructed from the combined sample sets.

To analyze factors affecting the genetic relatedness of *M. kansasii* strains between pairs of persons from the study sample, we used hierarchical Bayesian spatial network methods implemented in the R package GenePair (*22*). As opposed to standard regression modeling, GenePair methods are designed to account for correlation observed in dyadic data given that the same person is represented across multiple pairs (i.e., network dependence), spatial correlation between paired outcomes because of unmeasured transmission dynamics, and specific distributional features of genetic relatedness outcomes (*23*).

We modeled 2 outcomes of genetic relatedness: a binary outcome in which we classified pairs of strains as clustered or not based on a threshold SNP distance and a continuous measure of SNP distance between pairs of strains. For the binary outcome, the bimodal distribution of observed SNP distances between strains (Appendix Figure 1) suggested a threshold of <45 SNPs to classify pairs of strains as being within a cluster. In a sensitivity analysis, we also investigated a more conservative cutoff of <32 SNPs. We fit all models using Markov chain Monte Carlo sampling techniques and assessed convergence using trace plots and Geweke’s diagnostic for all relevant model parameters. When making statistical inference, we reported posterior means and 95% equal-tailed quantile-based credible intervals.

Independent variables included the time between the date of each participant’s sputum collection in days, the combined age of both participants in years, the age difference between pairs of participants in years, whether both were male or female, whether either person in the pair had cavitary disease on chest radiograph, geographic distance between participant households in kilometers, whether participant households were both supplied by the same water purification plants, whether both participants resided in villages with a high degree of linkage to the 4 heavy industrial zones, and whether the patients had received their diagnosis and been treated in the same hospital. We chose demographic and clinical variables included in the models a priori on the basis of clinical relevance. 

The study was approved by the institutional ethics committees of the participating hospitals (KMUHIRB-F(I)-20210173, KMUHIRB-E(I)-20210380). The study funders had no role in the design and conduct of the study, the analysis and interpretation of data, or in the preparation, review, or approval of the manuscript.

## Results

### Demographics

Of the 216 cases with complete information, the mean patient age was 67 years (SD 17.6); 146 (67.6%) were men and 70 (32.4%) women (Table). Mean body mass index was 21 (SD 4.1). Of the 216 case-patients, 46 (21.3%) were current smokers, 45 (20.8%) were ex-smokers, and 125 (57.9%) had never smoked. On chest radiograph, 42 (20.1%) persons had consolidation, 37 (17.1%) had fibrocavitary disease, 81 (38.8%) had nodular bronchiectasis, 3 (1.4%) had nodules, 1 (0.5%) had fibrosis, and 52 (24.1%) had mixed image patterns. Tuberculosis had been diagnosed previously in 52 (24.1%) persons.

**Table:** Demographics of included population in study of phylogeographic analysis of *Mycobacterium kansasii* isolates from patients with *M. kansasii* lung disease in industrialized city, Taiwan*

Characteristic	Value
Total no. participants	216
Mean age, y (SD)	67.29 (17.59)
Sex	
F	70 (32.4)
M	146 (67.6)
Mean body mass index (SD)	20.98 (4.14)
Smoking status	
Current smoker	46 (21.3)
Ex-smoker	45 (20.8)
Never smoked	125 (57.9)
Chest radiograph findings	
Consolidation	42 (20.1)
Fibrocavitary disease	37 (17.1)
Fibrosis	1 (0.5)
Nodular bronchiectasis	81 (38.8)
Nodule	3 (1.4)
Other†	52 (24.1)
Prior tuberculosis	52 (24.1)
Chronic obstructive disease	51 (23.6)
Pulmonary disease	
Bronchiectasis	35 (16.2)
Asthma	19 (8.8)
Pneumoconiosis	4 (1.9)
Lung cancer	9 (4.2)
Inhaled corticosteroid use	19 (8.8)
Residential water purification plant‡	
Baolai	1 (0.5)
Weng Park	22 (10.2)
Lingkou	4 (1.9)
Lujhu	1 (0.5)
Kaotan	99 (45.8)
Fongshan	70 (32.4)
Chengcinghu	128 (59.3)
Pingding	84 (38.9)

*Values are no. (%) except as indicated. †Other patterns included mixtures of the aforementioned patterns. ‡Participant residences may be supplied by >1 water purification plant.

### Sources of Residential Water Purification

Participant households were served by a median of 2 (maximum 3) of 8 different water purification plants. Chengcinghu supplied 128 (59.3%) of participant households, Pingding supplied 84 (38.9%), Kaotan supplied 99 (45.8%), Fongshan supplied 70 (32.4%), Weng Park supplied 22 (10.2%), Lingkou supplied 4 (1.9%), and Baolai and Lujhu each supplied 1 (0.5%) (Table; Figure 1).

### Factors Associated with Genetic Relatedness between Pairs of Participants

**Figure 2:**
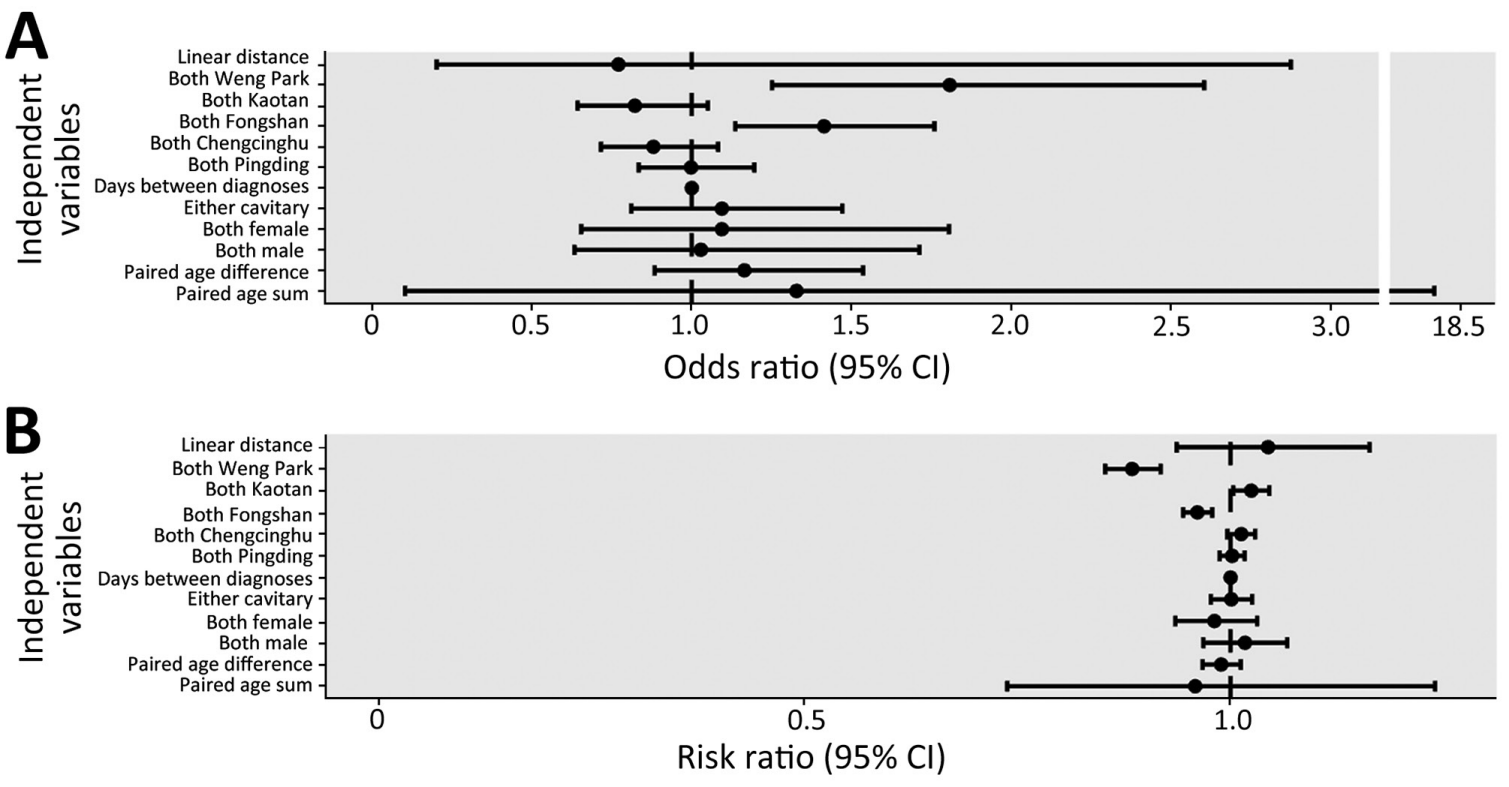
Associations of environmental and clinical risk factors with genetic relatedness based on pair-level data using hierarchical Bayesian regression models in phylogeographic analysis of *Mycobacterium kansasii* isolates from patients with *M. kansasii* lung disease in industrialized city, Taiwan. A) Odds ratios for pairs of *M. kansasii* isolates to be in a genetic cluster (using the single-nucleotide polymorphism [SNP] cutoff of 45). An odds ratio of >1 suggests that the risk factor was associated with genetic clustering. B) Risk ratios for increase in SNP distance between pairs of isolates. A risk ratio of <1 suggests that the risk factor was associated with a shorter pairwise SNP distance. The 3 smaller water purification plants (Lingkou, Baolai, and Lujhu) were not considered in the analysis as they together only provided service to 6 participants.

Before analyzing genetic relatedness of *M. kansasii* strains, we masked areas of horizontal sequence transfer as previously described. This process reduced the median number of SNPs per strain from 23 (interquartile range [IQR] 537) to 13 (IQR 14.25). We constructed hierarchical Bayesian models to evaluate factors associated with being part of a genetic cluster (i.e., *M. kansasii* SNP distance between isolates of <45) as well as factors associated with continuous SNP distance. The model results (Figure 2) indicated that for each pair of participants, if both participant residences were supplied by the Weng Park water purification plant, their *M. kansasii* isolates had increased likelihood of being genetically clustered (odds ratio [OR] 1.81, 95% credible interval [CrI] 1.25–2.60), after adjusting for the other relevant factors. This finding was mirrored in the SNP model, where the SNP distances between the *M. kansasii* isolates of those pairs of persons were ≈12% smaller on average (risk ratio [RR] 0.88, 95% CrI 0.85–0.92). With a clustered model and a more conservative cutoff of 32 SNPs, the magnitude of the association was similar but not statistically significant (OR 1.54, 95% CrI 0.96–2.45) (Appendix Figure 2). We found a lower magnitude of association for the Fongshan water purification plant with a clustering OR of 1.39 (95% CrI 1.12−1.71) and a RR of SNP distance of 0.96 (0.94–0.98). We observed an inverse but statistically insignificant association between linear spatial distance and odds of clustering; adjusted OR was 0.77 (95% CrI 0.20–2.88) for every 1 km increase in spatial distance. Having a linkage to the same heavy industrial zone and sharing the same healthcare facility were not significantly associated with genetic relatedness, nor did they significantly alter the effect sizes for other variables in the model. Therefore, we removed those 2 variables from the final multivariable models for parsimony.

The statistical models also provided an estimate of a spatially referenced random effect parameter for each person that described the participants’ residual (after adjustment for other factors) risk of being infected with an *M. kansasii* isolate that was genetically similar to other participants in the study. We mapped posterior mean estimates for those parameters (Appendix Figure 3). From visual inspection, we noted no obvious areas of increased residual risk.

### Tree Structure of *M. kansasii* Population

**Figure 3:**
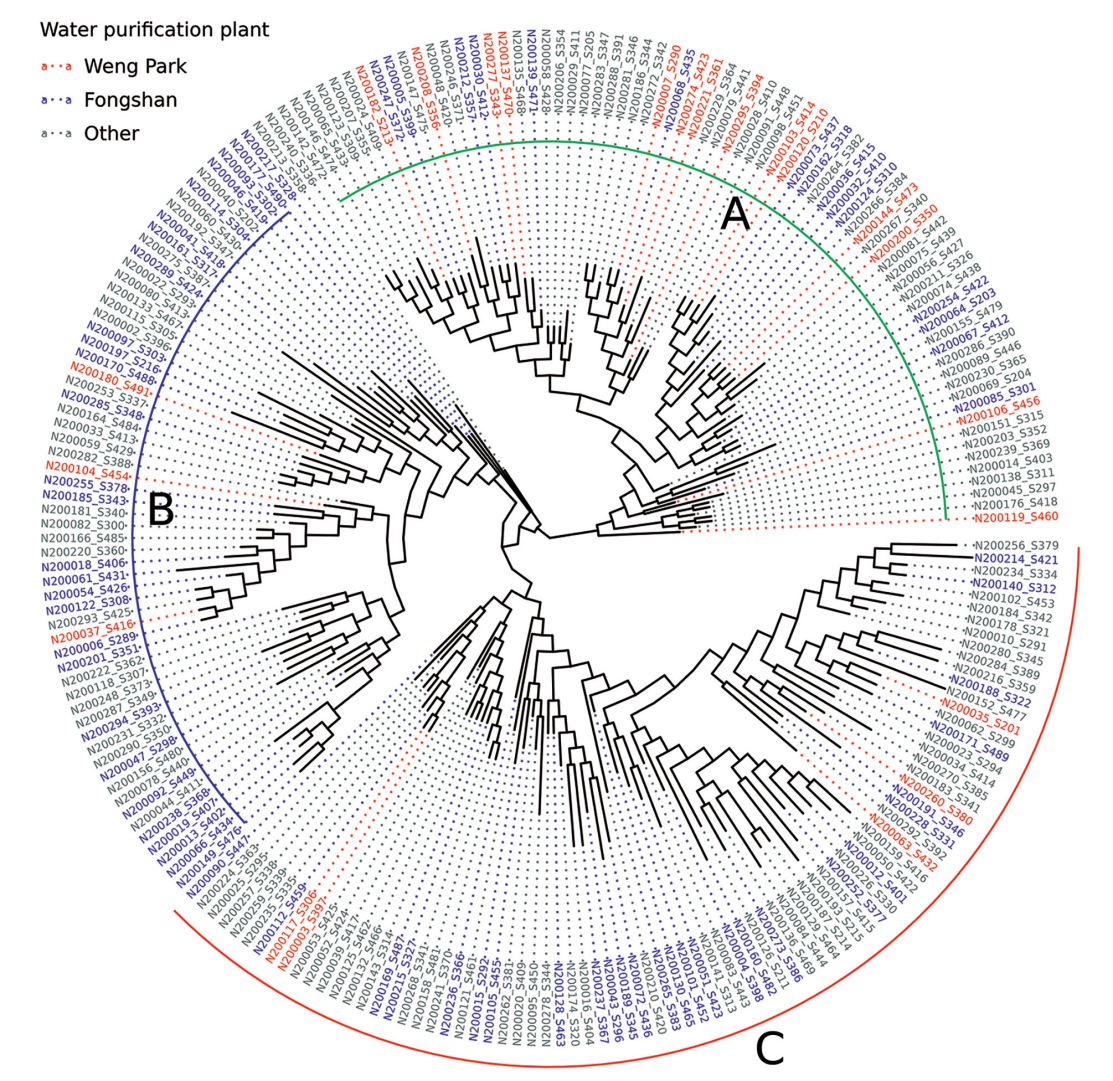
Maximum-likelihood phylogram of clinical *Mycobacterium kansasii* isolates from patients with *M. kansasii* lung disease in industrialized city, Taiwan. Phylogeny with major clades are labeled as A, B, and C; colored text indicates source of water for patient households.

We created a maximum-likelihood phylogram that showed 3 main clades (Figure 3). Participants with residential water supplied by the Weng Park water purification plant were more likely to be in clade A. Of the 73 persons in clade A, 14 (19.2%) were supplied by Weng Park. Of 58 participants in clade B, 3 (5.2%) had water purified by Weng Park; 5 (6.1%) of 82 participants in clade C had water purified by Weng Park (p = 0.008 for the association between genetic clade and Weng Park). Participants supplied by the Fongshan water purification plant were more likely to be in clade B. Of the 58 participants in clade B, 27 (46.6%) were supplied by Fongshan. Of 73 participants in clade A, 15 (20.5%) were supplied by Fongshan; 27 (32.9%) of 82 in clade C were supplied by Fongshan (p = 0.006 for the association between genetic clade and Fongshan). We conducted a sensitivity analysis that included an additional 22 publicly available *M. kansasii* isolates from outside Taiwan in the phylogenetic analysis. The resulting phylogeny revealed the 3 major clades, which contained the same Taiwan samples as in the main analysis (Appendix Figure 4). Similarly, when we constructed a phylogeny of our samples from Taiwan without masking recombinant regions, the same clades were identified with identical members in clades A and B. Clade C in the phylogeny constructed without recombination masking contained all of the samples seen in the primary analysis but also overlapped with 59 samples from clade B.

## Discussion

Using densely sampled cases of *M. kansasii* infection from a tropical metropolitan city in southern Taiwan, we investigated environmental factors associated with genetic relatedness using WGS data. We found that pairs of persons with *M. kansasii* lung disease living in a district served by specific water purification plants (Weng Park and Fongshan) were at greater risk of being infected with genetically similar *M. kansasii* isolates. The association of smaller spatial distances between pairs of persons had a statistically significant trend toward more genetically similar *M. kansasii* strains.

We previously conducted a spatial analysis of 537 *M. kansasii* cases from 4 major tertiary hospitals in Kaohsiung and identified 2 suspicious spatial clusters (*11*). In this analysis, we found that the Weng Park and Fongshan water purification plants, both near 1 of the previously identified spatial hotspots, were associated with increased genetic relatedness among case pairs.

Weng Park, the water purification plant with the strongest signal of genetic relatedness (adjusted OR 1.81, 95% CrI 1.25–2.60) among *M. kansasii* cases, accounted for only 4% of the total water supply in the study area but was associated with 22 (10%) of the total 216 cases. We also observed that Weng Park was significantly associated with 1 major *M. kansasii* clade in our phylogenic analysis. Because water from different water plants mixed in underground water pipes, determining the source of supply for a particular household was difficult. Given the relatively low number of households supplied by Weng Park, the cases and households labeled as Weng Park might have also received their supply from other water plants, such as Kaotan and Pingding (Figure 1). The misclassification of water purification plants at the household level would lead us to underestimate the association between Weng Park and genetic relatedness of *M. kansasii* among case pairs.

This study does not provide a mechanism for the association between Weng Park and Fongshan water plants and the genetic clustering of *M. kansasii*–infected cases. Each water plant applied different methods of water purification, resulting in differences in pH value or organic matter content that might affect the risk for contamination and growth of *M. kansasii* (*6*). Different sources of raw water might also potentially influence the microbiological ecology; a 2003 analysis found that water from Weng Park had substantially higher general hardness than other water plants, suggesting a higher contribution from underground water as the source (*13*). The presence of more sediment accumulation in pipelines from underground water than from surface water might accelerate biofilm development (*24*). As an example of the effect of the water treatment and distribution system on NTM abundance, a previous study in the United States reported low NTM relative abundances in Mississippi River water, a source for the drinking water system, but high relative abundances in the distribution system and tap water (*25*). Further environmental samplings should be conducted to examine the distribution of *M. kansasii* isolates in different water sources and plants, particularly Weng Park and Fongshan water plants.

Recent analyses of *M. abscessus*, another pathogenic NTM, have suggested that transmission might occur from person-to-person, especially in cystic fibrosis patients who might attend the same clinic (*5*). Our study did not find a potential link between shared clinic visits and the genetic clustering of *M. kansasii* in the investigated cases by analyzing pairs of persons’ strains treated at the same hospital.

Although WGS has been widely applied to understand the transmission dynamics of *M. tuberculosis*, its application for studying NTM transmission including *M. kansasii* is still limited (*9*,*26*,*27*). The population structure and genomic diversity of *M. kansasii* on a global scale have been previously reported (*28*), but the genomic diversity over a well-defined geography has not been characterized. We used the cutoff of 45 and 32 SNPs to define genetic relatedness on the basis of the empirical SNP distribution in our population, but the relationship between those measures of genetic distance and transmission remains uncertain. A major difference between *M. tuberculosis* and *M. kansasii* is that genomic recombination occurs frequently in *M. kansasii* through distributive conjugal transfer (*28*). A recent study using 60 samples of *M. kansasii* from different provinces of China revealed that the pairwise SNP distance (after masking recombinant regions) of those isolates were all within 20 SNPs and suggested a threshold of 4 SNPs to define clustering (*29*). Both that study and ours excluded recombinant regions using the same methods, further complicating the interpretation of our analysis in terms of transmission inference. In addition, selecting an effective threshold to cluster cases with a potential shared exposure site might be dependent on the local epidemiology and genetic diversity of the tested population. Along with this study, 2 other large analyses of clinical *M. kansasii* whole-genome sequences (*9*,*29*) showed that, after masking recombinant regions, the resulting sequences appeared to belong to homogeneous clusters with maximum SNP distances on the order of 100 SNPs. The largest analysis to date created a phylogeny that estimated the most recent common ancestor of a global collection of clinical isolates to be timed to the early 1900s (*9*).

A major strength of this study is the combination of WGS and detailed spatial information on the environmental determinants of interest, including the water supply and heavy industrial zoning. The novel Bayesian hierarchical modeling approach correctly accounts for the correlation of pairwise spatial-genetic data and the simultaneous adjustment for potential confounders. Previous studies on the transmission of *M. kansasii* have mostly applied conventional genotyping methodologies (e.g., restriction fragment length polymorphism or targeted PCR analysis) (*30*,*31*).

The first limitation of this study is that the *M. kansasii* isolates came from 1 major tertiary medical center and its affiliated hospitals (accounting for 56% of all *M. kansasii* isolates from all major medical centers in Kaohsiung during the same period) (*11*). *M. kansasii* infection is not a notifiable disease in Kaohsiung, and thus the population coverage of this analysis is not comprehensive. The suboptimal population coverage posed a challenge in identifying environmental exposures. Second, our analysis revealed an association between certain water purification plants (Weng Park and Fongshan) and genetic relatedness, but the route of transmission cannot be confirmed without environmental sampling. Weng Park only accounted for 10% of total cases and Fongshan accounted for 32% of cases. Environmental determinants of most *M. kansasii* cases remain to be elucidated. Third, we only have crude (district-level) spatial coverage of the water purification plants, and misclassification of water supply at the household level might well occur. Last, we used the residential address as the proxy for exposure assessment, and we were not able to obtain definitive workplace exposures.

In conclusion, our novel spatial phylogenetic analysis in a densely sampled, well-defined geography revealed an independent association between certain water purification plants and the genetic relatedness of *M. kansasii* isolates. Our approach demonstrated the utility of combining WGS sequencing and readily available clinical and environmental information to obtain useful insights into the transmission of *M. kansasii* to trigger further environmental investigation and control measures.

Appendix Additional information about phylogeographic analysis of *Mycobacterium kansasii* isolates from patients with *M. kansasii* lung disease in industrialized city, Taiwan
