# Supplementary material for: Phylogeographic Analysis of Mycobacterium kansasii Isolates from Patients with M. kansasii Lung Disease in Industrialized City, Taiwan
Source: Emerg Infect Dis. 2024 Aug;30(8):1562–70. doi: 10.3201/eid3008.240021 (PMC11286038; doi:10.3201/eid3008.240021)
Supplement: Appendix — Additional information about phylogeographic analysis of Mycobacterium kansasii isolates from patients with M. kansasii lung disease in industrialized city, Taiwan [file 24-0021-Techapp-s1.pdf]

Article DOI: https://doi.org/10.3201/eid3008.240021

EID cannot ensure accessibility for supplementary materials supplied by authors. Readers who have difficulty accessing supplementary content should contact the authors for assistance.

Phylogeographic Analysis of *Mycobacterium kansasii* Isolates from Patients with *M. kansasii* Lung Disease in Industrialized City, Taiwan

Appendix

Supplementary methods

Mycobacterial isolation and species identification

We collected pre-treatment respiratory samples from bronchoalveolar lavage fluid or sputum before ***M. kansasii*** lung disease was diagnosed. We inoculated all samples on both Mycobacteria Growth Indicator Tube (MGIT, BD Biosciences, Sparks, MD, USA) and Löwenstein–Jensen(L-J) agar. Species identification was performed with Matrix-assisted laser desorption/ionization-time of flight (MALDI-TOF) spectrometry analyses from colonies isolated on L-J solid culture medium using the Bruker Biotyper system with Mycobacteria Library v2.0 (Bruker Daltonics GmbH & Co. KG., Bremen, Germany). We extracted genomic DNA from each collected isolate following the cetyltrimethylammonium bromide (CTAB) protocol (*1*). The DNA samples with sufficient quantity and an OD260/OD280 ratio of 1·5 or higher were stored at −20°C until sequencing.

WGS classification and filtering

We removed 22 samples with most reads from a species other than M. kansasii from analysis. One additional sample had a large degree of non-mycobacterial reads, and 4 samples were from participants missing clinical or residential data. We excluded these from analysis, leaving 216 samples for our primary analysis. For M. kansasii samples with more than 5% of

reads identified as non-M. kansasii (as determined by bracken), we included a filtering step with Kraken Tools to remove non-mycobacterial reads. These filtered samples had a range of non-mycobacterial reads of 5 to 38% before filtering.

Analysis of genetic relatedness

The unit of analysis was the pair of individuals from the study sample. The goal was to assess the associations between pair-level genetic relatedness and its potential determinants including environmental exposure, spatial distance, demographics, and clinical features (investigated at the individual and pair level). For the binary outcome of clustering, we used a logistic regression framework to estimate the associations between individual-level and pair-wise variables, and genetic clustering. For the count valued outcome of SNP distance, we used negative binomial regression. Our Markov chain Monte Carlo sampling used standard weakly informative prior distributions specified for the model parameters. In total we collected 110,000 MCMC samples from the algorithms, with a burn-in of 10,000 states, and thinned by a factor of 10 to reduce posterior autocorrelation.

Analysis on heavy industry exposure and genetic relatedness of M. kansasii

As described in the introduction section, we hypothesized that specific water supplies or heavy industrial zones might be associated with the risk of M. kansasii infection. To explore the potential association between heavy industry exposure and genetic relatedness of ***M. kansasii***, we collected geographic information on heavy industrial zoning in Kaohsiung from the Open Data Platform of Kaohsiung City Government. Since people only work in the industrial zones during the day and we did not have information on specific work addresses, we used a probabilistic approach to identify study participants with a high probability of working in one of the four (Linhai, Dafa, Fongshan, and Linyuan) heavy industrial zones in Kaohsiung. In brief, we used the aggregated mobility information inferred from mobile phone calling data to identify villages with the highest day-time connectivity to each of the four heavy industrial zones. We determined whether each of the study participants are connected to each of the four industrial zones or not based on their residential village.

Following our approach in analyzing the association between potential risk factors and genetic relatedness, we conducted our analysis for two outcomes of genetic relatedness: 1) a binary outcome where we classified pairs of strains as in the same cluster based on a threshold

SNP distance and 2) a continuous measure of SNP distance between pairs of strains. We applied the hierarchical Bayesian spatial network methods to estimate the association between heavy industrial zoning and genetic relatedness (see the methods section for details). In the hierarchical Bayesian regression analysis based on paired data, there was no independent significant association between heavy industrial zoning (based on residential address among districts in the top 5% linkage to each industrial area) and risk of genetic relatedness after adjusting for other covariates for both binary and continuous genetic measures.

References

1. Larsen MH, Biermann K, Tandberg S, Hsu T, Jacobs WR Jr. Genetic manipulation of *Mycobacterium tuberculosis*. Curr Protoc Microbiol. 2007;10:10A.2. **PMID 18770603**

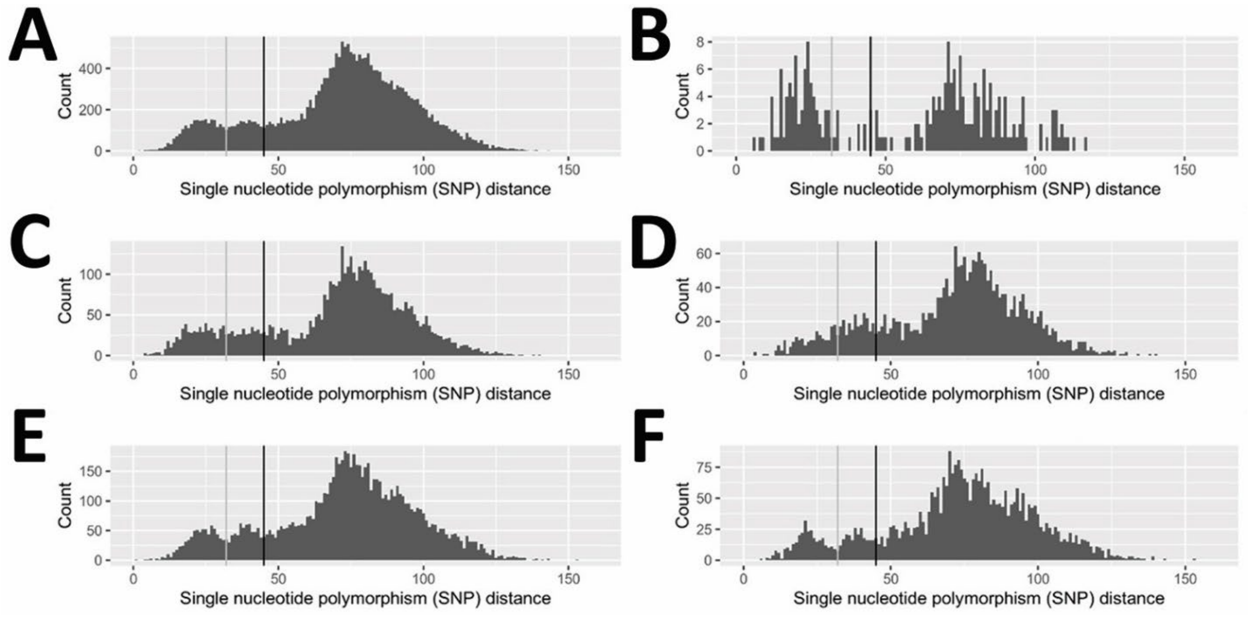

Appendix Figure 1. Single nucleotide polymorphism (SNP) distances between pairs of isolates, stratified by water supply to residential addresses of participants. Black line indicates cluster cutoff of 45 SNPs. Grey line indicates cluster cutoff of 32 SNPs. Count indicates the number of participants pairs with that SNP distance between their isolates.

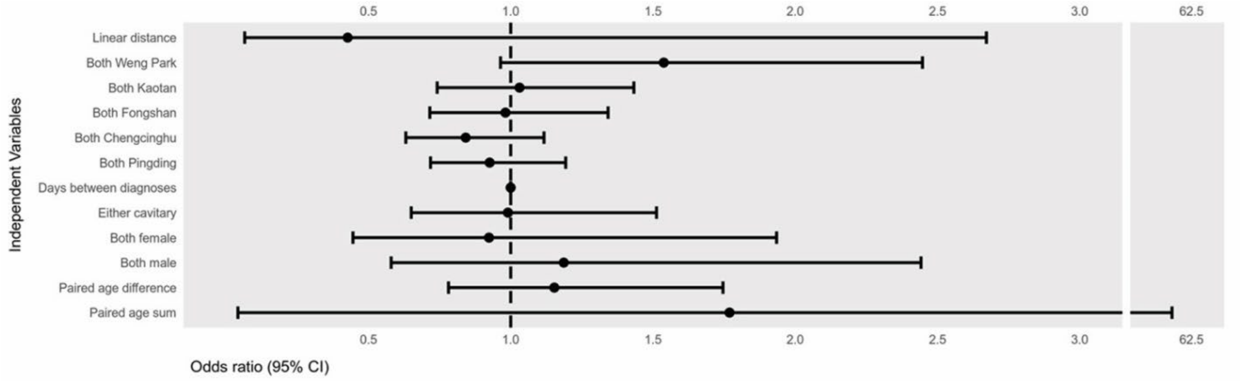

Appendix Figure 2. Cluster model (32 SNP cutoff) estimates.

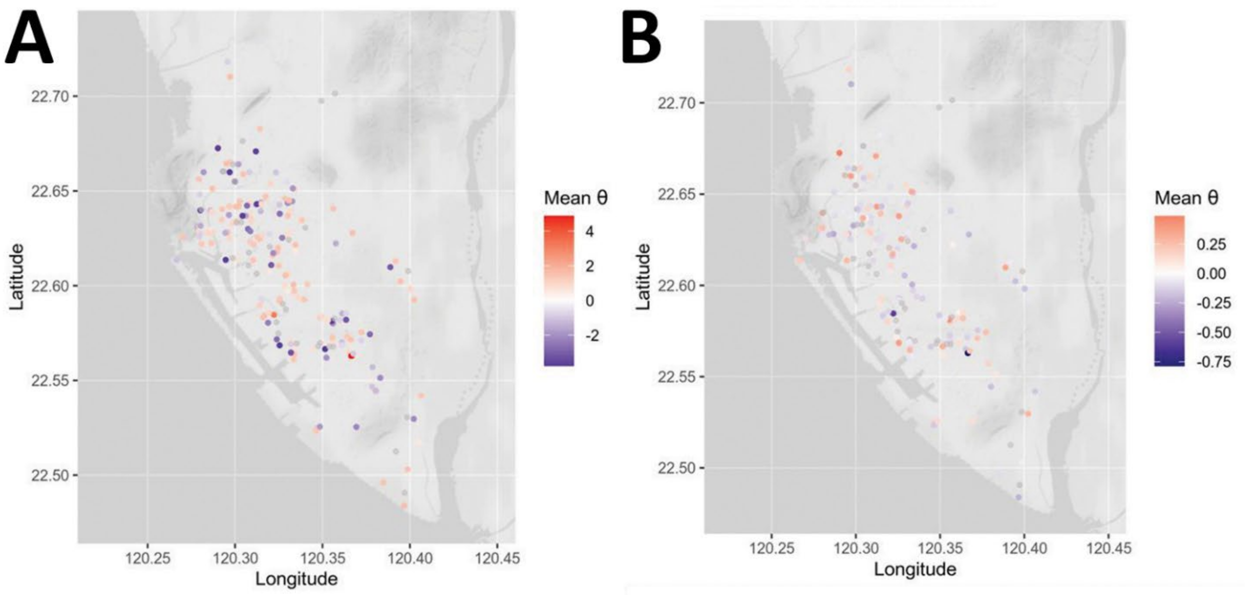

Appendix Figure 3. Participant level random effects (θ). A) Cluster model estimates, B) SNP model estimates. Participant home locations (jittered) covered by individual level random effects value (if significant)

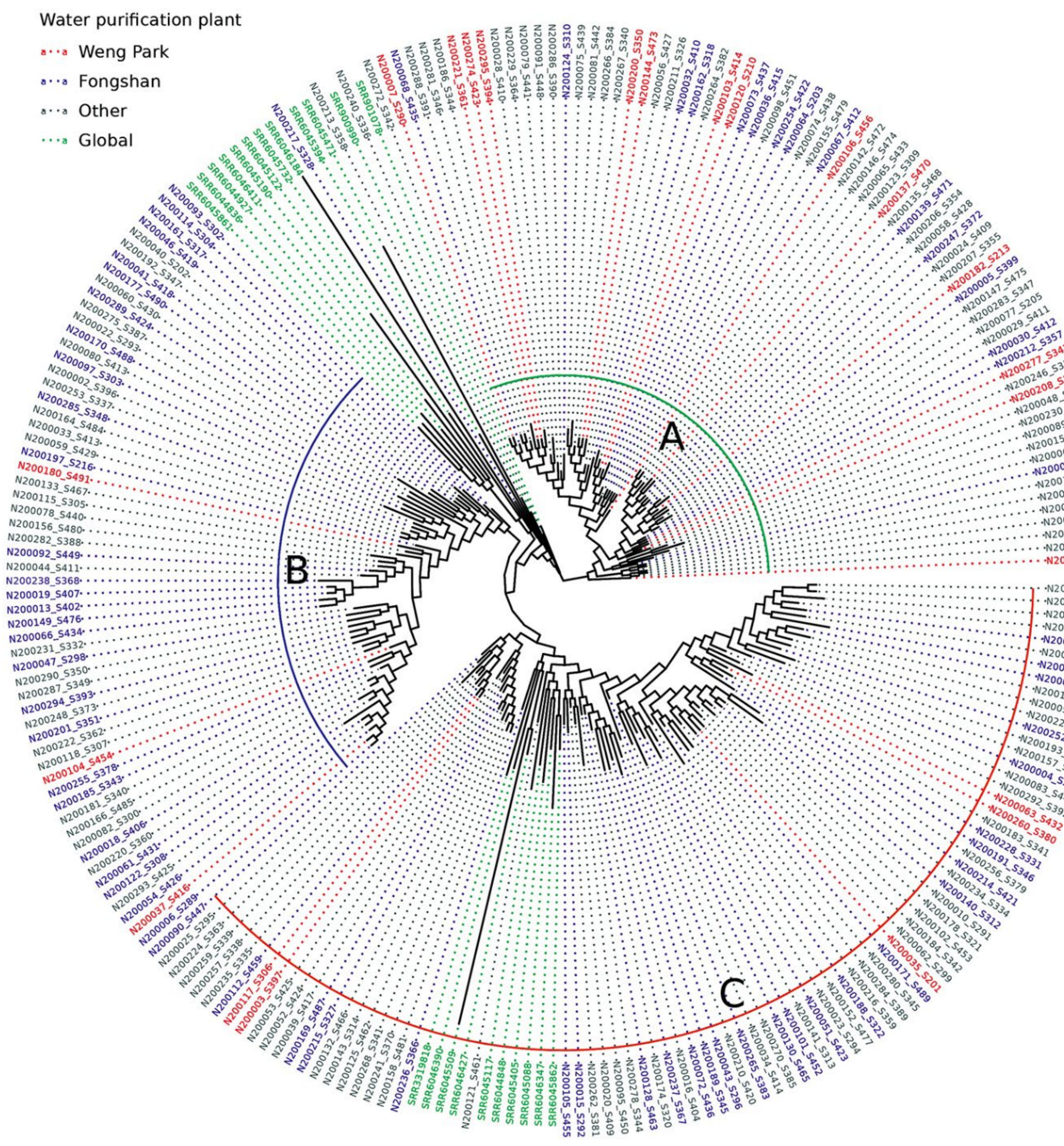

Appendix Figure 4. Phylogeny of *M. kansasii* isolates including global samples.
